# Defect size and surgical site are key predictors of surgical site infection risk in dermatologic surgery: A retrospective cohort study

**DOI:** 10.1016/j.jdin.2024.09.017

**Published:** 2024-11-19

**Authors:** Rawlings E. Lyle, Michelle Vy, Mehrnaz Mehrzad, Daniel B. Eisen

**Affiliations:** a Department of Dermatology, University of California, Davis, School of Medicine, Sacramento, California; b Department of Dermatology, University of California, Davis, Sacramento, California

**Keywords:** dermatologic surgery, epidemiology, general dermatology, infection, Mohs surgery, oncology, reconstruction, surgical site infection

**Figure:**
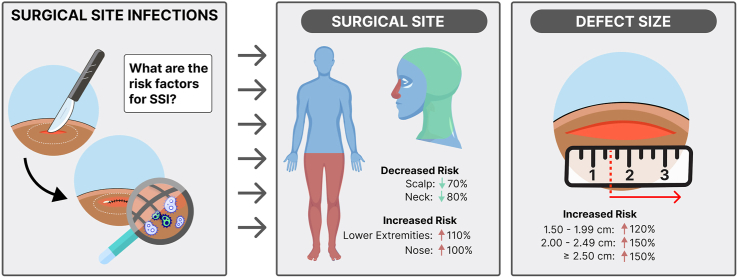

*To the Editor:* Despite over 50 years of prevention efforts, the rate of surgical site infections (SSIs) has remained relatively unchanged, with dermatological surgeries reporting a range of incidence between 0.7% and 4% depending on factors such as anatomical site and closure technique.^1^ In contrast, antibiotic usage in dermatology as a whole has declined by more than one-third, while usage in dermatologic surgery has increased by nearly 70%— highlighting ongoing challenges in SSI management and the importance of identifying modifiable risk factors.^2^

To improve our understanding, we conducted a retrospective cohort study at UC Davis Medical Center, reviewing 2411 Mohs micrographic and excisional surgeries from January 2016 to December 2019. For Mohs surgery, we use chlorhexidine for all sites except near the eyes or ears, where betadine or alcohol (for allergies) is used. The same disinfection protocol applies to excisions, adjusted based on surgical site. To identify SSI, we used the Center for Disease Control-National Healthcare Safety Network 2023 clinical indicators^3^ and postoperative antibiotic prescriptions, rather than relying solely on culture results.

This study focused on modifiable risk factors, specifically defect size and closure technique. Patient demographics, comorbidities, and detailed surgical information formed the basis of our logistic regression analysis, conducted in R (version 4.3.2) with methods published to Posit Cloud.^4^ Further details are in Supplementary Fig 1, available via Mendeley at https://data.mendeley.com/datasets/3yh38zt95f/1

Our findings show that closure technique did not significantly correlate with SSI rates (Fig 1). However, defect sizes ≥1.50 cm were significantly associated with increased SSI risk (*P* ≤ .01). Infection rates by defect size are included in the Supplementary Fig 2, available via Mendeley at https://data.mendeley.com/datasets/3yh38zt95f/1. Surgeries of the nose and lower extremity showed higher SSI risks (*P* values: .03 and .01, respectively), while procedures on the neck and scalp exhibited reduced incidence (*P* values: .04 and .03, respectively). Other risk factors, such as smoking history, prophylactic antibiotic use, diabetes, and immunosuppression, did not significantly impact SSI incidence (*P* > .05).^5^ Infection data by patient and surgical characteristics can be found in Table I and Supplementary Table I, available via Mendeley at https://data.mendeley.com/datasets/3yh38zt95f/1.

**Fig 1:**
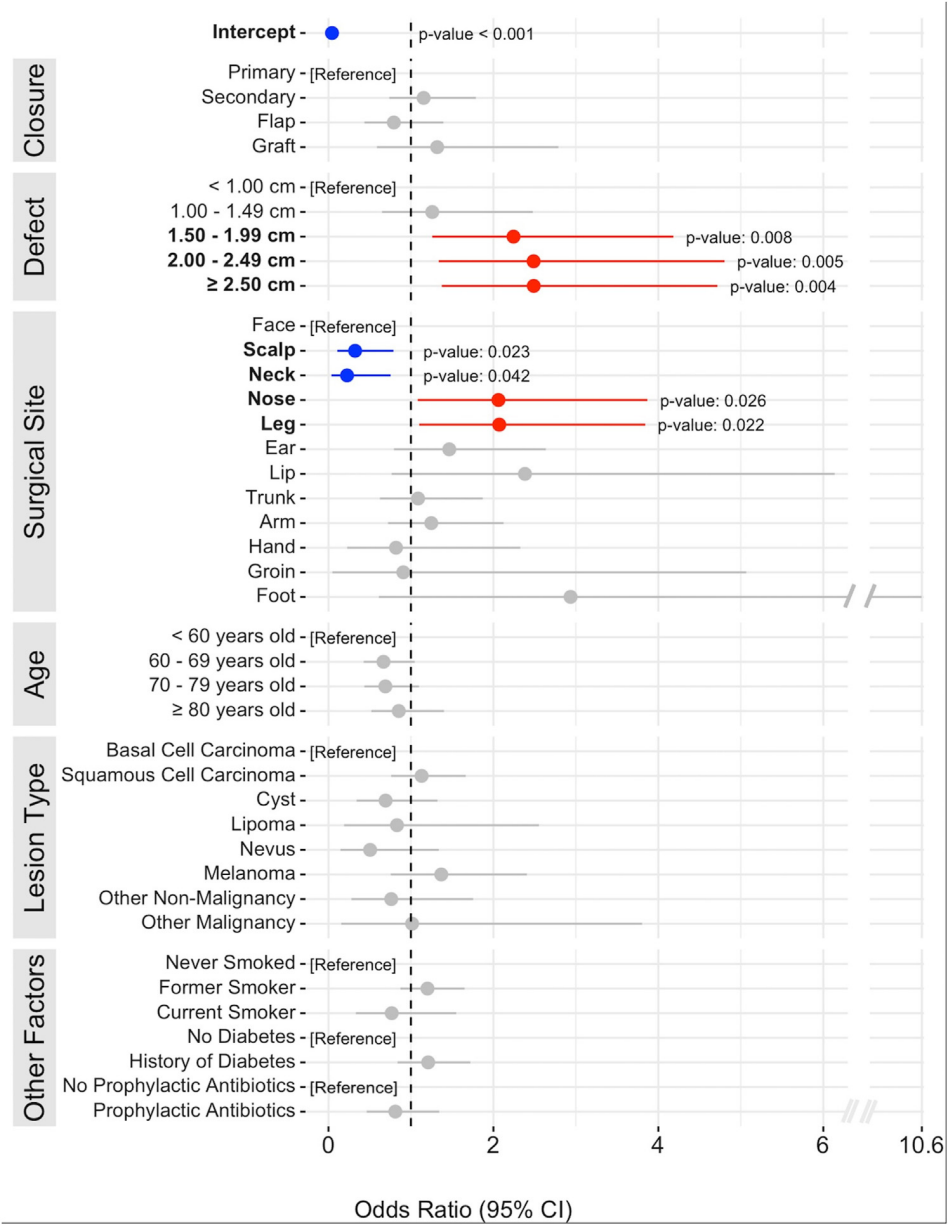
Forest plot of demographic and surgical factors affecting SSI risk, highlighting significant increases (*red*) and decreases (*blue*) in risk. Includes odds ratios and 95% confidence intervals. *SSI*, Surgical site infection.

**Table I:** Patient characteristics by closure technique and infection

	Total	Closure technique				Infection	No infection
	*n* = 2411	Primary	Secondary	Flap	Graft	*n* = 188 (8.5%)	*N* = 2223 (91.5%)
		*n* = 1533	*n* = 502	*n* = 288	*n* = 88		
Age, y							
Mean (SD)	68 (13)	66 (13)	71 (12)	71 (12)	73 (10)	69 (13)	68 (13)
Median	69					70	69
<60	520 (21.6)	401 (26.2)	66 (13.1)	47 (16.3)	6 (6.8)	41 (21.8)	479 (21.5)
60-69	735 (30.5)	485 (31.6)	145 (28.9)	75 (26.0)	30 (34.1)	51 (27.1)	684 (30.8)
70-79	706 (29.3)	412 (26.9)	170 (33.9)	96 (33.3)	28 (31.8)	51 (27.1)	655 (29.5)
≥80	450 (18.7)	235 (15.3)	121 (24.1)	70 (24.3)	24 (27.3)	45 (23.9)	405 (18.2)
Gender, *n* (%)							
Female	988 (41.0)	666 (43.4)	182 (36.3)	112 (38.9)	28 (31.8)	89 (47.3)	899 (40.4)
Male	1423 (59.0)	867 (56.6)	320 (63.7)	176 (61.1)	60 (68.2)	99 (52.7)	1324 (59.6)
Smoking history, *n* (%)							
Never	1319 (54.7)	870 (56.8)	264 (52.6)	139 (48.3)	46 (52.3)	98 (52.1)	1221 (54.9)
Current	142 (5.9)	567 (37.0)	212 (42.2)	134 (46.5)	37 (42.0)	8 (4.3)	134 (6.0)
Former	950 (39.4)	96 (6.3)	26 (5.2)	15 (5.2)	5 (5.7)	82 (43.6)	868 (39.0)
Diabetes mellitus history, *n* (%)							
None	1903 (78.9)	1228 (80.1)	388 (77.3)	219 (76.0)	68 (77.3)	141 (75.0)	1762 (79.3)
Present	508 (21.1)	305 (19.9)	114 (22.7)	69 (24.0)	20 (22.7)	47 (25.0)	461 (20.7)
Prophylactic antibiotics, *n* (%)							
None	2195 (91.0)	1408 (91.8)	459 (91.4)	255 (88.5)	73 (83.0)	171 (91.0)	2024 (91.0)
Prescribed	216 (9.0)	125 (8.2)	43 (8.6)	33 (11.5)	15 (17.0)	17 (9.0)	199 (9.0)

By broadening the clinical criteria for SSI identification, our methodology better reflects clinical practice, resulting in a higher infection rate than typically reported. Of the 188 infections, 46% (86) had positive cultures, 4% (8) had negative cultures, and 50% (94) had no culture collected. Clinically suspicious infections treated with antibiotics were considered significant, reflecting real-world practice. Defect size and anatomical location were more predictive of SSI risk than closure technique. Smaller sample sizes for flaps and grafts may explain variability in infection rates in these groups. We also acknowledge that procedural duration, not captured in our data set, may contribute to infection rates as a confounding factor. The lack of observed benefit from prophylactic antibiotics raises questions about their routine use in dermatologic surgery.

Given the single-center nature of our study, further multicenter research is warranted to confirm these findings and their applicability to broader clinical practice. Nonetheless, our findings suggest it is important to reevaluate current strategies for assessing SSI risk.

## Conflicts of interest

None disclosed.

## References

[1] Hanly A.M., Daniel V.T., Mahmoud B.H. (2021). Controversies in defining a surgical site infection following Mohs micrographic surgery: a literature review. J Am Acad Dermatol.

[2] Barbieri J.S., Bhate K., Hartnett K.P., Fleming-Dutra K.E., Margolis D.J. (2019). Trends in oral antibiotic prescription in dermatology, 2008 to 2016. JAMA Dermatol.

[3] Prevention CDC (2023).

[4] Lyle R.E. (2023). UCD dermatologic surgery SSI. https://posit.cloud/content/8152382.

[5] Schlager J.G., Hartmann D., Ruiz San Jose V., Patzer K., French L.E., Kendziora B. (2022). Procedure-related risk factors for surgical site infection in dermatologic surgery. Dermatol Surg.

